# The effect of 12 weeks of aerobic exercise training with or without saffron supplementation on diabetes‐specific markers and inflammation in women with type 2 diabetes: A randomized double‐blind placebo‐controlled trial

**DOI:** 10.1002/ejsc.12125

**Published:** 2024-06-14

**Authors:** Ali Rajabi, Ali Akbar Nezhad Gharehlo, Elham Madadizadeh, Aref Basereh, Kimya Khoramipoor, Hossein Pirani, Karen Khoramipour, Othmar Moser, Kayvan Khoramipour

**Affiliations:** ^1^ Faculty of Educational Sciences and Psychology Department of Exercise Physiology University of Mohaghegh Ardabili Ardabil Iran; ^2^ Faculty of Physical Education and Sport Sciences Department of Exercise Physiology University of Tehran Tehran Iran; ^3^ Faculty of Physical Education Department of Exercise Physiology Shahid Bahonar University Kerman Iran; ^4^ Department Exercise Physiology Kharazmi University Tehran Iran; ^5^ Faculty of Nursing and Midwifery Department of Nursing Kurdistan University of Medical Sciences Kurdistan Iran; ^6^ Faculty of Marine Sciences Department of Science Maritim University of Chabahar Chabahar Iran; ^7^ Faculty of Humanities and Social Sciences Department of Sport Science Kurdistan University Kurdistan Iran; ^8^ Exercise Physiology and Metabolism (Sports Medicine) BaySpo—Bayreuth Centre of Sports Science University of Bayreuth Bayreuth Germany; ^9^ Interdisciplinary Metabolic Medicine Trials Unit Medical University of Graz Graz Austria; ^10^ Neuroscience Research Center Institute of Neuropharmacology Kerman University of Medical Sciences Kerman Iran

**Keywords:** aerobic training, FIB, HCY, IL‐6, obesity, saffron, TNF‐*α*, type 2 diabetes

## Abstract

This study was conducted to investigate the effects of 12 weeks of aerobic exercise (AT) and saffron supplementation on hemostasis, inflammatory markers, and insulin resistance in obese women diagnosed with type 2 diabetes (T2D). A total of 44 women with T2D (mean age: 54.12 ± 5.63 years, mean BMI: 31.15 ± 1.50 kg/m^2^, HbA1c: 85 ± 4.2 mmol/mol) were included in a randomized, double‐blind, placebo‐controlled study. We were randomly assigned to one of four groups (*n* = 11 per group): saffron + training (ST), placebo + training (PT), saffron supplement (SS), and placebo (P). The ST and PT groups completed 12 weeks of AT (three sessions per week of mild to moderate intensity). The ST and SS groups were administered a daily dose of 200 mg of saffron powder for 12 weeks. Fasting blood samples were collected 48 h before the first AT session and/or nutritional supplementation and 48 h after the last AT session and/or nutritional supplementation. Post‐evaluation, homeostatic model assessment of insulin resistance value (HOMA‐IR, *p* < 0.001) and serum levels of glucose (*p* < 0.001), fibrinogen (FIB, *p* < 0.001), homocysteine (HCY, *p* < 0.001), interleukin‐6 (IL‐6, *p* < 0.001), and tumor necrosis factor *α* (TNFα, *p* < 0.001) showed significant reduction in the ST, PT, and SS groups compared to the P group (*p* < 0.05). In particular, the ST group showed a more significant reduction in all variables compared to the PT and SS groups (*p* < 0.05). Our results suggest that a 12‐week intervention with AT and saffron supplementation can independently improve markers related to hemostasis, inflammation, and insulin resistance. However, their combination showed the greatest effectiveness on the above markers.

## Highlights

- Type 2 diabetes can disturb hemostasis and induce inflammation.
- 12 weeks of aerobic training (AT) and saffron supplementation can independently improve hemostasis, inflammation, and insulin resistance.
- The combination of 12 weeks of AT and saffron supplementation showed the greatest effectiveness on hemostasis, inflammation, and insulin resistance.

## 1 INTRODUCTION

Cardiovascular diseases (CVDs) represent the primary cause of global mortality, claiming approximately 17.9 million lives each year. In 2008, CVDs were responsible for 30% of all global deaths, a figure projected to escalate, causing over 23 million deaths by 2030. Low‐ and middle‐income countries bear a substantial burden, accounting for more than 80% of CVD‐induced fatalities (Wang et al., 2016).

Risk factors for CVDs encompass age, gender, hyperglycemia (diabetes), an unhealthy diet, obesity, and physical inactivity (Khoramipour et al., 2020). Notably, individuals with type 2 diabetes (T2D) face an 80% higher mortality rate due to CVDs (Alzahrani & Ajjan, 2010; Khoramipour, Bejeshk, et al., 2023). Besides hyperglycemia (Rajizadeh et al., 2023), individuals with T2D often experience an imbalanced hemostatic state, further exacerbating cardiovascular complications. Fibrinogen (FIB) emerges as a pivotal hemostatic risk factor, influencing platelet aggregation, plasma viscosity, and fibrin formation. Elevated FIB levels, prevalent in women and escalating after menopause, significantly contribute to CVDs. In diabetes, heightened FIB may result from hyperreactive platelets (Zhang et al., 2018).

Homocysteine (HCY) is an additional factor implicated in cardiovascular issues due to its detrimental impact on cardiovascular endothelium and smooth muscle cells (Li et al., 2020). Elevated HCY levels (hyperhomocysteinemia) intensify thrombogenicity, oxidative stress, and endothelial dysfunction, correlating with insulin resistance, dyslipidemia, and inadequate disease control in diabetes (Li et al., 2020; Shidfar et al., 2011).

Another aspect of the complex interplay involving CVD, T2D, and obesity is the escalation of inflammatory cytokines, notably interleukin‐6 (IL‐6) and tumor necrosis factor alpha (TNF‐α) within the serum (Ebrahimnezhad et al., 2023; Saberi et al., 2024). Elevated levels of IL‐6 contribute to heightened thrombotic events by increasing FIB. Furthermore, increased TNF levels have been associated with thrombosis. IL‐6 and TNF exert inhibitory effects on the expression of glucose transporter type 4 (GLUT‐4), potentially promoting insulin resistance when their levels rise. Consequently, reducing IL‐6 and TNFα levels in T2D patients is proposed to ameliorate their condition and alleviate symptoms of CVD (Rajabi et al., 2022; Zhou et al., 2020).

Several chemical supplements have exhibited the potential to reduce inflammation and enhance hemostatic regulation. However, due to considerable side effects, healthcare professionals are increasingly inclined towards the use of organic supplements with similar effects. Saffron, recognized for diverse medical applications, stands as a highly favored organic supplement. Saffron, scientifically known as Crocus sativus L. and belonging to the Iridaceae family, comprises crucial compounds such as aldehydes (picrocrocin and safranal) and flavonoids (crostin and crocin) (Hosseinzadeh & Nassiri‐Asl, 2013). Studies have demonstrated the efficacy of saffron supplementation in treating CVD (Pourmasoumi et al., 2019) and improving diabetes by activating the AMP‐activated protein kinase (AMPK)/GLUT‐4 pathway (Dehghan et al., 2016). Additionally, saffron possesses anti‐inflammatory properties (Hosseinzadeh & Younesi, 2002).

Exercise training is another regulator of inflammation and homeostasis (Khajehlandi et al., 2021; Madadizadeh & Aminaei, 2022; Orumiyehei et al., 2022; Rajizadeh et al., 2023; Rami et al., 2022; Rezaei et al., 2023). In addition, The American Diabetes Association recommends individuals with DT2 engage in 2–3 exercise sessions per week targeting major muscle groups. This should include at least 150 min of moderate‐intensity aerobic exercise per week, or at least 90 min of high‐intensity aerobic exercise per week (Feinman et al., 2015). Various exercise protocols have been studied for their effects on serum levels of FIB, HCY, IL‐6, and TNFα in both healthy and diabetic participants (e Silva et al., 2020). For instance, an 8‐week high‐intensity interval training (HIIT) resulted in decreased serum FIB levels among men with T2D (Hoseinrezaie et al., 2022; Rezaeimanesh, 2020). Conversely, a 10‐week study reported an increase in FIB concentration in both resistance training and aerobic training (AT) groups (Banz et al., 2003). Additionally, a 16‐week aerobic and resistance training did not significantly affect plasma HCY levels in patients with T2D (e Silva et al., 2020).

Considering the potential combined effects of saffron and exercise training, we aimed to investigate the impact of a 12‐week AT and saffron supplementation on hemostasis, inflammation, and insulin resistance in post‐menopausal obese women diagnosed with T2D.

## 2 MATERIALS AND METHODS

This study was a randomized, double‐blind, placebo‐controlled trial. The participants were 44 middle‐aged obese menopausal women, with an average age of 54.12 ± 5.63 years and a BMI of 31.15 ± 1.50 kg/m^2^, all diagnosed with T2D and residing in Kermanshah Province, Iran. The selection process involved convenience sampling, adhering to specific inclusion criteria. These criteria encompassed the absence of cardiovascular and musculoskeletal disorders, glycosylated hemoglobin (HbA1c) levels below 9.9% (85 mmol/mol), no diabetic complications (neuropathy, nephropathy, or retinopathy), no regular AT, non‐smoking status, diabetes duration less than 5 years, and a maximum intake of one type of oral anti‐diabetic tablet daily. However, participants would be excluded from the study if any of the following conditions were observed: refusal to participate in the exercise programs, onset of acute illness during the study period, engagement in additional physical exercises, consumption of other supplements, or non‐adherence to the supplement regimen. Additionally, participants would be excluded if diagnosed with cardiovascular or musculoskeletal diseases, experienced sudden onset of diabetic complications (neuropathy, nephropathy, and retinopathy) during the study, initiated smoking, increased intake of more than one type of oral anti‐diabetic tablet at night, or underwent significant changes in the prescription of experimental drugs affecting blood sugar or lipid control. It is important to note that all participants were taking a consistent dosage of metformin and were not receiving insulin therapy. Participants with blood glucose levels exceeding 120 mg/dL (measured after a 12‐h fasting) were classified as diabetic (Khoramipour, Rezaei, et al., 2023; Rajizadeh et al., 2024; Xiao et al., 2024). The participants were allocated into four groups: saffron + training (ST) (*n* = 11), placebo + training (PT) (*n* = 11), saffron supplementation (SS) (*n* = 11), and placebo (P) (*n* = 11). The study's objectives and procedures were clearly elucidated, and written consent was obtained from all participants. During the course of the study, there were no noteworthy alterations in the prescription of medications to regulate glycemia or lipids, as indicated by the participants' physicians. The study received approval from the Ethics Committee of Mohaghegh Ardabili University (Ethics No: 1605/A. D; dated November 6, 2017).

### 2.1 Preparation and administration of saffron and placebo capsules

High‐performance liquid chromatography was used to analyze and determine the primary metabolites in saffron (Table S1). Empty gelatin capsules were purchased from the “Iran Capsule” company. These capsules do not contain preservatives or allergens and are of excellent quality. Powdered saffron, with a dose of 200 mg (in accordance with the identifier of the Food and Drug Organization of the Ministry of Health: 1021/50 and 111,191/50), is encapsulated and administered once daily at 6 p.m. for three months for both the ST and SS groups. 200 and 400 mg/day saffron supplementation usage in diabetic participants has also been reported in a previous study (Modaghegh et al., 2008). Parallel placebo capsules, mimicking the color and shape of saffron capsules, were formulated for the P and PT groups, containing 200 mg of wheat flour. Adherence to this regimen was emphasized for all participants, who were instructed not to take any medication except metformin during the study to control for potential confounding and interfering factors (Rajabi et al., 2022).

### 2.2 Participants' diet

Participants were asked not to consume any kinds of saffron or saffron‐included nutrients. The nutritional data acquisition involved utilizing a 24‐h dietary recall method. This entailed recording dietary intake for two non‐holiday weekdays and one weekend day to compute the mean nutrient intake. Participants were instructed to provide a detailed account of all foods and beverages consumed within the past 24 h during each reporting period. Each participant completed this dietary recall questionnaire a total of 30 non‐consecutive times, amounting to three times a week throughtout the study (Hosseini Esfahani et al., 2010; Willett, 1998). The data related to participants' diets has been provided in the supplementary file (Table S2).

### 2.3 Exercise intervention training program

The AT program, conducted three days per week, was administered to both the PT and ST groups, comprising three components for each session: a 10‐minute warm‐up (self‐paced running), the main part, and a 10‐minute cool‐down (self‐paced running). Due to the participants' lack of regular AT and limited physical fitness, the main part during the initial week entailed 20 min of AT an intensity ranging from 40% to 45% of the target heart rate (THR). The THR was computed using Karvonen's formula: THR = [(MHR − RHR) × %Intensity] + RHR, where MHR represents the maximum heart rate and RHR stands for the resting heart rate. The intensity was increased by 5% every 2 weeks until the final week. Additionally, the training duration was increased by 5 min each week until reaching 50 min by the seventh week, after which it remained constant until the 12th week (see Table 1). Participants' heart rates were meticulously monitored using a Polar watch during the AT sessions (Polar Electro Oy Professoriate 5, Polar, US6584344, FI‐90440 Kempele, Finland). The AT regimen comprised versatile movements such as aerobic dancing.

**TABLE 1:** AT protocol.

Week	Intensity (%HRmax)	Duration (minute)
1	40–45	20
2	40–45	25
3	50–55	30
4	50–55	35
5	55–60	40
6	55–60	45
7	60–65	50
8	60–65	50
9	65–70	50
10	65–70	50
11	70–75	50
12	70–75	50

### 2.4 Measurement of variables

To assess body weight, BMI, and body fat percentage, a body composition analyzer (In Body version 3.0, manufactured in Seoul, South Korea) was employed. Participants were required to stand on the device without any metallic objects and wear minimal clothing, ensuring contact with the device electrodes. Measurements were taken at least 3 h after breakfast.

Blood sampling involved obtaining a 10 cc venous blood sample from the left radial artery after a 12‐h fast. This was conducted in two stages: pre‐test (48 h prior to the first AT session) and post‐test (48 h after the last AT session).

FIB levels were quantified using a plasma coagulation auto analyzer (TOA Medical Electronics Model CA‐1000, USA) with a human FIB kit (Sigma Chemical Co., USA). HCY levels were assessed through enzyme immunoassay (EIA) utilizing a homocysteine kit (Axis‐Shield Diagnostics, UK). Serum levels of glucose, IL‐6, and TNFα were determined by measuring their optical density at 450 nm using an ELISA reader. The ELISA kits used were as follows: TNFα (Orgegium, Helsinki, Finland), IL‐6 (Boster, Pleasanton, CA, USA), and glucose (Pars Azmun, Tehran, Iran) (Basereh et al., 2017; Rahmaty et al., 2015).

Insulin resistance was gauged using the homeostatic model assessment for insulin resistance (HOMA‐IR), calculated utilizing the following formula:

HOMA‐IR = fasting blood glucose (mmol/L) _ fasting insulin (_u/mL)/22.5.

### 2.5 Statistical analysis

Descriptive statistics are presented as the mean and standard deviation. Normality and homogeneity of data were assessed using the Shapiro–Wilk and Levene tests, respectively. A two‐way analysis of variance (ANOVA) was employed to ascertain the primary group (e.g. ST, PT, SS, P) and time (pre‐/post‐test), as well as their potential interaction. In cases of significant main effects or interactions, the Bonferroni post‐hoc test was conducted to delineate differences among the groups. Statistical analysis was carried out using GraphPad Prism version 9, with a significance level set at ***p*** < 0.05.

## 3 RESULTS

There was no significant difference between groups in age, height, and duration of disease (Table S3) as well as nutrient uptake (TABLE S2). At pre‐test, there were no differences in all variables between groups (*p* > 0.05). Two‐way ANOVA showed a significant interaction (Group × Time) for body weight (F_(3,80)_ = 11.6, *p* < 0.001), BMI (F_(3,80)_ = 10.18, *p* < 0.001), and fat percentage (F_(3,80)_ = 8.98, *p* < 0.001) (Table 2). There was a significant decrease in body weight in the PT (*p* = 0.001), ST (*p* = 0.012), and SS group (*p* = 0.007) when compared against the P group. In addition, there was a significant decrease in body fat percentage in the PT (*p* = 0.001), ST (*p* = 0.002), and SS (*p* = 0.003) groups when compared against the P group. There was a significant decrease in BMI in the PT (*p* = 0.000), ST (*p* = 0.001), and SS (*p* = 0.001) groups when compared to the P group (Table 2).

**TABLE 2:** Participants' anthropometric properties (Mean ± SD).

Variables	Groups	Pre‐test	Post‐test
Body weight (kg)	ST	79.12 ± 4.35	75.50 ± 5.45^a^
	PT	85.87 ± 3.30	79.15 ± 4.1^a^
	SS	82.47 ± 6.91	77.8 ± 6.89^a^
	P	85.12 ± 5.32	88.19 ± 4.98
Body fat (%)	ST	30.98 ± 3.19	26.43 ± 2.52^a^
	PT	31.12 ± 2.87	29.62 ± 1.96^a^
	SS	32.12 ± 3.50	30.69 ± 3.52^a^
	P	35.62 ± 2.26	36.02 ± 2.40
BMI (kg/m^2^)	ST	31.02 ± 2.24	27.97 ± 2.68^a^
	PT	32.25 ± 1.05	29.40 ± 1.94^a^
	SS	31.27 ± 3.65	31.1 ± 3.57^a^
	P	35.30 ± 3.63	35.15 ± 3.75

Abbreviations: P, Placebo; PT, placebo + training; SS, Saffron + supplementation; ST, saffron + training.

^a^ Significant decrease in the ST, PT; and SS groups compared to the P group.

**FIGURE 1:**
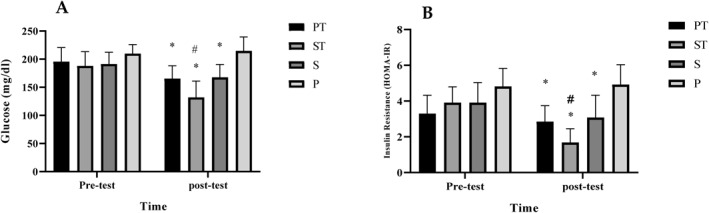
Glucose levels in the pre‐test and post‐test. *Significant decrease (*p* < 0.001) in the ST, PT, and SS groups compared to the P group. # significant difference (*p* < 0.001) between ST with PT and SS (A), Insulin levels in the pre‐test, and post‐test. *Significant decrease (*p* < 0.001) in the ST, PT, and SS groups compared to the P group. # significant difference between ST with PT and SS (B). P, Placebo; PT, placebo + training; SS, Saffron + supplementation; ST, saffron + training.

**FIGURE 2:**
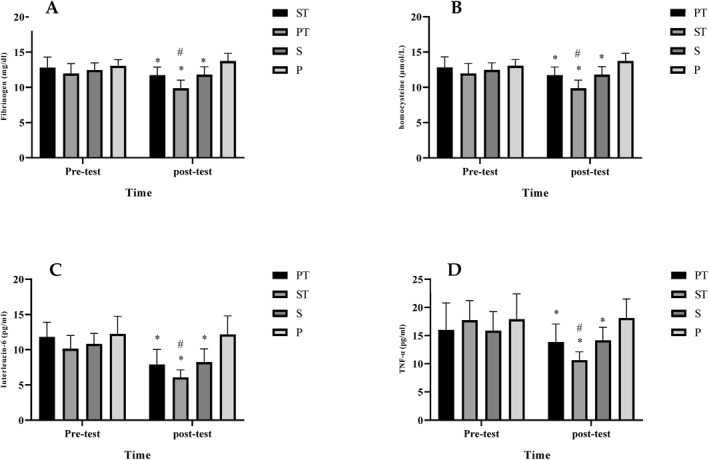
FIB levels in the pre‐test and post‐test. *Significant decrease (*p* < 0.001) in the ST, PT, and SS groups compared to the P group. # (*p* < 0.001) significant difference between ST with PT and SS (A), HCY levels in the pre‐test, and post‐test. *(*p* < 0.001) Significant decrease in the ST, PT, and SS groups compared to the P group. # significant difference (*p* < 0.001) between ST with PT and SS (B), IL‐6 levels in the pre‐test, and post‐test. *Significant decrease (*p* < 0.001) in the ST, PT, and SS groups compared to the P group. # significant difference (*p* < 0.001) between ST with PT and SS (C), tumor necrosis factor α levels in the pre‐test, and post‐test. * Significant decrease (*p* < 0.001) in the ST, PT, and SS groups compared to the P group. # significant difference (*p* < 0.001) between ST with PT and SS (D). P, Placebo; PT, placebo + training; SS, Saffron + supplementation; ST, saffron + training.

At pre‐test, there were no differences in all variables between groups (*p* > 0.05). The results of two‐way ANOVA showed a significant interaction between groups and time for glucose (F _(3,80)_ = 12.11, *p* < 0.001) (Figure 1A), insulin (F _(3,80)_ = 8.02, *p* < 0.001) (Fig 1B), FIB (F _(3,80)_ = 12.03, *p* < 0.001) (Figure 2A), HCY (F _(3,80)_ = 9.42, *p* < 0.001) (Fig 2B), IL‐6 levels (F _(3,80)_ = 16.10, *p* < 0.001) (Fig 2C), and TNFα levels (F _(3,80)_ = 6.98, *p* < 0.001) (Fig 2D). There was a significant decrease in all variables in the PT, ST, and SS groups when compared against the P group with the most reduction in the ST group (*p* < 0.05).

## 4 DISCUSSION

In this study, our objective was to examine the impact of 12 weeks of saffron supplementation and AT on hemostasis, inflammation, and insulin resistance in middle‐aged obese women diagnosed with T2D. Our findings demonstrated that both 12 weeks of AT and saffron supplementation alone significantly affected serum levels of glucose, insulin resistance, FIB, HCY, IL‐6, and TNFα in diabetic patients. However, the combined approach yielded even more substantial improvements. Blood glucose levels and insulin resistance notably decreased in the ST, PT, and SS groups. This suggests that both AT and saffron supplementation can influence blood glucose levels, likely by stimulating AMPK signaling effectively as mentioned by Park et al. (Park et al., 2020). This stimulation led to enhanced GLUT‐4 translocation into the cell membrane, increased glucose uptake, and improved insulin sensitivity (Park et al., 2020). A study by Tajaddini et al. (2023), and Milajerdi et al. (2018), showed that administering 100 and 15 mg/day of saffron supplementation to diabetic patients can improve glycemic status, lipid profile, and oxidative status. Additionally, serum levels of FBS, insulin, and HOMA‐IR were significantly improved. Samarghandian et al. (Samarghandian et al., 2014) also confirm that saffron extract can improve insulin resistance and glucose absorption by peripheral tissues.

Furthermore, our study revealed that while both AT and saffron supplementation reduced serum IL‐6 levels, the combination of these interventions resulted in even more pronounced improvements. Our findings are in line with another study (Lage & Cantrell, 2009), which demonstrated a significant reduction in serum IL‐6 and TNFα levels in the PT, SS, and ST groups. The antioxidant properties of saffron likely contributed to this reduction, as saffron can diminish levels of reactive oxygen species, such as hydrogen peroxide, by inhibiting oxidative stress, a pivotal factor in pro‐inflammatory marker production (Lage & Cantrell, 2009; Tofighi & Ghafari, 2013). Recent studies (Akbari‐Fakhrabadi et al., 2019; Asdaq & Inamdar, 2010) reported that the saffron components, including crocin, crocetin, and safranal, all showed antioxidant properties, while their combination as saffron enhanced their effectiveness (Akbari‐Fakhrabadi et al., 2021). In addition, Peeri et al. (Peeri et al., 2012) proposed that a combination of saffron intake (25 mg/kg) and treadmill running could effectively reinforce the antioxidant system in T2D. Zamani et al. (2022) also attributed the anti‐inflammatory effect of saffron to reducing oxidative stress.

While some studies reported no significant changes in IL‐6 levels after AT (Lage & Cantrell, 2009), another observed decreased inflammatory and increased anti‐inflammatory cytokines in individuals with T2DM following AT (Tofighi & Ghafari, 2013). In addition to reducing oxidative stress, the reduction of body fat, which is a potential source of inflammatory cytokine production, is considered another explanation for reducing inflammation after AT (Kohut et al., 2006).

The current study also revealed a reduction in serum FIB levels after 12 weeks of AT and saffron supplementation, with a more favorable outcome observed with the combination. Various mechanisms may explain this effect, including alterations in neurotransmitters and catecholamines (Rezaimanesh & Amiri‐Farsani, 2011), modifications in fibrinolytic system activity, decreased FIB production by the liver, changes in BMI, lipid profile, and plasma FIB concentrations (Myint et al., 2008). In our study, 12 weeks of AT potentially decreased FIB by reducing cytokine activity and minimizing FIB formation in liver cells. Additionally, an increase in epinephrine and norepinephrine may have contributed, as these neurotransmitters are known to influence plasma FIB concentrations (Rezaimanesh & Amiri‐Farsani, 2011). Numerous studies have established a direct relationship between FIB and changes in body fat, body weight, and BMI (Nath et al., 2019).

Furthermore, HCY levels were found to decrease in the ST, PT, and SS groups compared to the P group, consistent with Alomari et al. (Alomari et al., 2016) which showed that participants with higher levels of physical activity had less HCY than participants with lower levels of physical activity. In contrast, other studies reported that AT could either maintain or increase HCY levels (e Silva et al., 2020; Herrmann et al., 2003). Herman et al. (Herrmann et al., 2003) compared three types of acute endurance exercises (marathon, 100‐km run, and 120‐km mountain bike race) and observed that running a marathon increased HCY by 64%, possibly due to exercise‐induced changes in blood concentration. On the other hand, Silva et al. (e Silva et al., 2020) demonstrated that 16 weeks of AT and resistance training performed twice a week did not significantly change plasma HCY levels in individuals with T2D. The low frequency of training (two sessions per week) might explain the lack of effectiveness in this study.

## 5 CONCLUSION

Our findings indicate that a 12‐week intervention involving AT and saffron supplementation can independently enhance indicators associated with hemostasis, inflammation, and insulin resistance. Notably, the combined approach demonstrated the most significant efficacy in impacting these markers. Nonetheless, further original research is needed to fully elucidate this relationship.

## AUTHOR CONTRIBUTIONS

Conceptualization, Ali Rajabi, Ali Akbar Nezhad Gharehlo, Elham Madadizadeh, Aref Basereh, Kimya Khoramipoor, Hossein Pirani, Karen Khoramipour, Othmar Moser, and Kayvan Khoramipour; writing—original draft preparation, Ali Rajabi, Ali Akbar Nezhad Gharehlo, Elham Madadizadeh, Aref Basereh, Kimya Khoramipoor, Hossein Pirani, and Karen Khoramipour; writing—review and editing, Othmar Moser and Kayvan Khoramipour. All authors have read and agreed to the published version of the manuscript.

## CONFLICT OF INTEREST STATEMENT

The authors declare no conflict of interest.

## INSTITUTIONAL REVIEW BOARD STATEMENT

The animal study protocol was approved by the Institutional Review Board of Mohaghegh Ardabili University (Ethics No: 1605/A. D; date November 6, 2017).

## INFORMED CONSENT STATEMENT

Informed consent was obtained from all subjects involved in the study.

## Supporting information

Supporting Information S1

## Data Availability

Upon reasonable request the data will be provided by the corresponding author.
