# Supplementary material for: The effect of 12 weeks of aerobic exercise training with or without saffron supplementation on diabetes‐specific markers and inflammation in women with type 2 diabetes: A randomized double‐blind placebo‐controlled trial
Source: Eur J Sport Sci. 2024 Jun 14;24(7):899–906. doi: 10.1002/ejsc.12125 (PMC11235750; doi:10.1002/ejsc.12125)
Supplement: Supplementary file 1 — Supporting Information S1 [file EJSC-24-899-s001.docx]

**Supplementary table 1.** Determination of the main metabolites of saffron**.**

| Safranal | Crocin | Picrocrocin | Name of the Substance |
| --- | --- | --- | --- |
| 26.1 | 16.2 | 14.8 | Retention time (minute) |
| 1.17 | 6.47 | 6.69 | Saffron metabolites levels (mg/g) |

**Supplementary table 2**. Participants’ nutrient intake (mean ± SD).

| Variable | ST | PT | SS | P |
| --- | --- | --- | --- | --- |
| Energy (calorie/day) | 1682.89 *±* 135 | 1692.36 *±* 166 | 1687.68 *±* 149 | 1705.71 *±* 125 |
| Energy (calorie/day) | 244.07 ± 62.48 | 225.93 ± 59.27 | 223.26 ± 44.76 | 252.84 ± 38.34 |
| Carbohydrate (g/day) | 244.07 *±* 62.48 | 225.93 *±* 59.27 | 223.26 *±* 44.76 | 252.84 *±* 38.34 |
| Protein (g/day) | 66.66 *±* 14.33 | 73.53 *±* 17.23 | 78.8 *±* 13.39 | 80.00 *±* 16.42 |
| Fat (g/day) | 45.33 *±* 12.78 | 48.33 *±* 13.87 | 44.05 *±* 14.11 | 46.08 *±* 11.13 |
| Fiber (g/day) | 12.75 *±* 3.75 | 13.24 *±* 4.45 | 12.92 *±* 5.11 | 14.18 *±* 4.21 |
| Calcium (mg/day) | 262.92 *±* 179.02 | 270.18 *±* 201.61 | 268.87 *±* 185.23 | 286.54 *±* 178.11 |
| Vitamin C (mg/day) | 55.69 *±* 25.12 | 59.77 *±* 26.24 | 57.26 *±* 27.65 | 62.97 *±* 29.44 |
| Vitamin E (mg/day) | 2.5 *±* 1.14 | 2.94 *±* 1.46 | 2.87 *±* 1.67 | 3.41 *±* 2.47 |
| Selenium (mg/day) | 42.90 *±* 23.26 | 48.81 *±* 21.67 | 47.89 *±* 22.54 | 53.65 *±* 24.11 |

ST: saffron + training group, PT: placebo + training group, SS: Saffron + supplementation group, and P: Placebo group.

**Supplementary table 3.** Confounding factors (mean ± SD).

| VARIABLE  GROUP | AGE | HEIGHT | Duration of diabetes (years) |
| --- | --- | --- | --- |
| ST | 53.27±6.18 | 162.90±3.67 | 3.9± 1.9 |
| PT | 55.72±6.32 | 163.18±2.78 | 4.5 ±1.0 |
| SS | 56.54±4.88 | 162.81±4.53 | 4.7 ±1.5 |
| P | 55.68±5.79 | 159.72±3.31 | 3.7± 1.4 |

ST: saffron + training group, PT: placebo + training group, SS: Saffron + supplementation group, and P: Placebo group.
